# A Comparison of Topical or Retrobulbar Anesthesia for 23-Gauge Posterior Vitrectomy

**DOI:** 10.1155/2014/237028

**Published:** 2014-11-18

**Authors:** Hande Celiker, Levent Karabas, Ozlem Sahin

**Affiliations:** ^1^Ophthalmology Clinic, Marmara University School of Medicine, 34899 Istanbul, Turkey; ^2^Ophthalmology Clinic, Kocaeli University School of Medicine, 41100 Kocaeli, Turkey

## Abstract

*Background*. To compare the efficacy and safety of topical anesthesia versus retrobulbar anesthesia in 23-gauge vitreoretinal surgery. *Materials and Methods*. A total of 63 patients scheduled for 23 G posterior vitrectomy without scleral buckling procedures were included in the study. The patients were randomly assigned to receive either topical (Group 1, *n* = 31) or retrobulbar anesthesia (Group 2, *n* = 32). Postoperatively, patients were shown a visual analogue pain scale (VAPS) from 1 (no pain or discomfort) to 4 (severe pain or discomfort) to rate the levels of pain. *Results*. There was more discomfort in patients in Group 2 while anesthetic was administered (Group 1:  1.0 ± 0, Group 2: 2.3 ± 0.7, *P* = 0.0001). Between the two groups the level of pain during surgery (Group 1: 1.4 ± 0.5, Group 2: 1.5 ± 0.5; *P* = 0.85) was noted. There was also no significant difference between two groups postoperatively (Group 1: 1.2 ± 0.4, Group 2: 1.3 ± 0.4; *P* = 0.28). There were no complications in either group related to the anesthetic technique. No patient needed sedation or anesthesia supplement during the surgery or postoperative period. *Conclusion*. Topical anesthesia in posterior vitrectomy procedures is an effective and safe method that is alternative to retrobulbar anesthesia.

## 1. Introduction

Local anesthesia techniques for vitreoretinal surgery include retrobulbar, sub-Tenon, peribulbar, and topical anesthesia [1–8]. Although the advantages of local anesthesia are well known and include more rapid return to ambulation for the patient, ability to perform outpatient procedure, avoidance of complications of general anesthesia, and quicker surgery, it has been the subject for some controversy [9–11]. Yepez et al. have shown that topical anesthesia combined with neuroleptic anesthesia was also safe and effective alternative to peribulbar or retrobulbar anesthesia in posterior vitrectomy procedures with 20 G technique with scleral buckling procedures [6]. There are also reports in the literature with topical technique with 23 G technique [12, 13]. One of them reported comparison of topical anesthesia and peribulbar anesthesia for 23 G posterior vitrectomy [13]. We think that retrobulbar anesthesia is a widespread method for the posterior ophthalmic procedures [3]. There is no prospective comparative study in the literature between the retrobulbar anesthesia and topical anesthesia for posterior 23 G transconjunctival sutureless vitrectomy.

## 2. Patient Selection

The research followed the tenets of the Declaration of Helsinki. Sixty-three patients undergoing posterior vitrectomy were enrolled in the study. Twenty-one-year and older patients were included if they had an indication for posterior vitrectomy without the need for scleral buckling. Patients were excluded from the study if they had previous posterior segment surgery, a known allergy to topical or retrobulbar anesthetic drugs, active ocular infection, nystagmus, claustrophobia, extreme anxiety, mental retardation, orthopnea, muscle spasm around the eye, or a problem with communication, such as a language barrier, deafness, dementia, or psychiatric illness. Patients were also excluded from the study if they expressed a preference for general anesthesia.

A block randomization schedule was generated by one of the study coinvestigators (LK) and kept confidential from the surgeon (HC). All patients signed informed consent forms after they had received an explanation of the nature and possible consequences of the procedure and had been given thorough preoperative counseling on what they would experience during surgery under topical anesthesia, including the possibility of some pain sensation in the eye.

## 3. Materials and Methods

This comparative prospective case series study was comprised of 63 patients scheduled for posterior vitrectomy using topical (31 eyes, Group 1) or retrobulbar (32 eyes, Group 2) anesthesia. Premedication included very low dosage of midazolam hydrochloride (0.3–1.0 mg). It was performed two hours before the surgeries to avoid the preoperative excitement. All of the patients were conscious and communicative throughout the procedure. No patient needed sedation, analgesia, or anesthesia supplement during the surgery or postoperative period. In the operating room, all patients received continuous nasal prong oxygen 4 l/minute, and baseline vital signs were obtained.

Before surgery, pupillary dilatation was obtained with 1% tropicamide and 5% phenylephrine. In Group 1 conjunctival cul-de-sac was initially anesthetized with 0.5% proparacaine hydrochloride drops three times in the fifteen minutes preceding surgery in the preoperative area, after 0.5% proparacaine hydrochloride absorbed four sponges were placed in the superior and inferior conjunctival culde-sac. They were removed after 15 minutes. The eye and surrounding area were cleaned and painted with povidone iodine 5%. After draping, speculum was inserted and one drop of anesthetic was administered and surgical procedure was begun. Anesthetic status was controlled by grasping the bulbar conjunctiva with colibri forceps [13]. One drop of anesthetic was applied every 30 minutes during the procedure.

Group 2 patients received approximately 4 to 6 mL of 0.5% bupivacaine and 2% prilocaine (1 : 1) into the retrobulbar space via a 27-gauge, 32-mm Atkinson needle.

While performing anesthesia and during the surgery, patients were asked to inform the surgeon whether they felt pain or not. All of the surgeries were done by the same surgeon (HC) in a standardized fashion (three-port double system pars plana entry and infusion cannula lower temporal area) using the associate surgical systems and 23-gauge surgical instruments (D.O.R.C. Dutch Ophthalmic Research Center; Zuidland, the Netherlands). Cataract surgery was performed if needed. A phaco-chop technique was used and foldable implants were inserted. At the conclusion of the case, the microcannulas were removed, and the sclerotomy sites were compressed with a cotton tip to allow self-sealing and adjust the conjunctiva to its original position. If any leakage occurred, defined as either bleb formation, tamponade escape, or unstable intraocular pressure measured digitally, a suture was placed at the site of leakage.

After surgery, patients were taken to the postoperative area where vital signs were obtained and the same observer (OS) collected patient assessment responses as soon as possible (Table 1). Questions were presented to the patients in a standardized written form. One hour after surgery each patient was shown a visual analogue pain scale (VAPS) with numeric and descriptive ratings from 1 (no pain and discomfort) to 4 (severe pain and discomfort) [6, 8, 13] again. Patients were asked to fill VAPS for the surgical conditions, pain during administration of anesthesia, pain during surgery, and postoperative pain. If patients were unable to see the scale or read the accompanying text, the scale was described and a score was obtained orally. The surgeon also completed a questionnaire on the surgical conditions, complications, and need for supplemental anesthesia.

Patients were evaluated on the first postoperative day and then at first week, second week, and first month. Best-corrected visual acuity, intraocular pressure, anterior chamber reaction, and fundus examination were noted.

Student *t*-test or *χ* ^2^ analysis was used for data comparison between the study groups. A *P* value less than 0.05 was considered significant.

## 4. Results

Of the 63 patients enrolled in the study, 31 were randomized to Group 1 and 32 were randomized to Group 2. Patients characteristics and indications for posterior vitrectomy were shown in Table 2.

**Figure 1:**
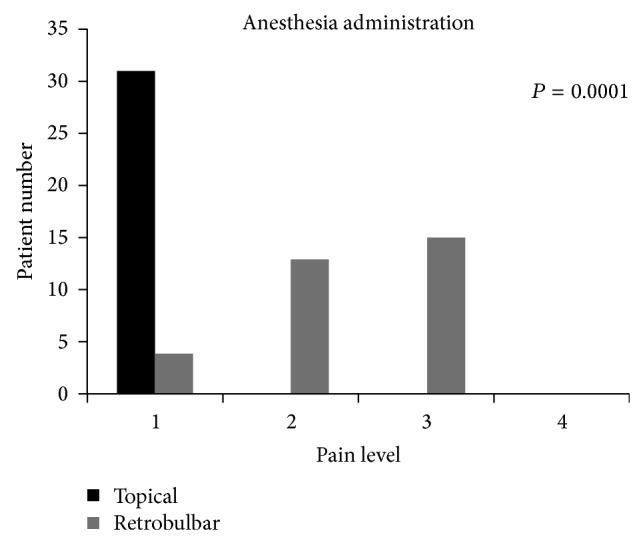
The pain scores recorded for delivery of anesthesia.

**Figure 2:**
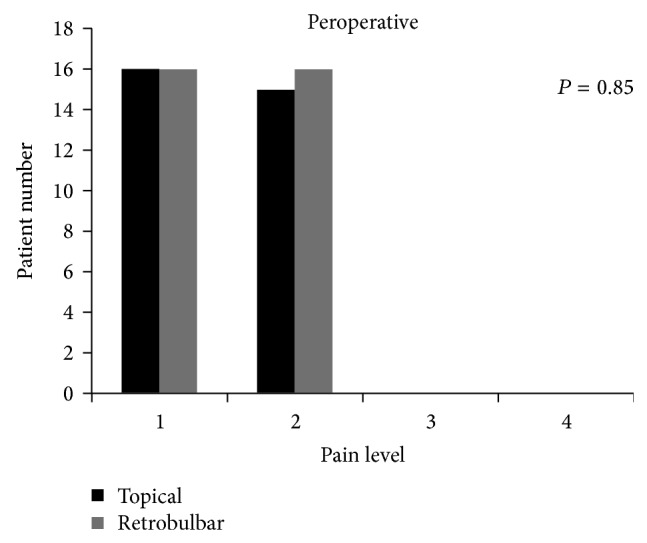
The pain scores recorded for surgery.

**Figure 3:**
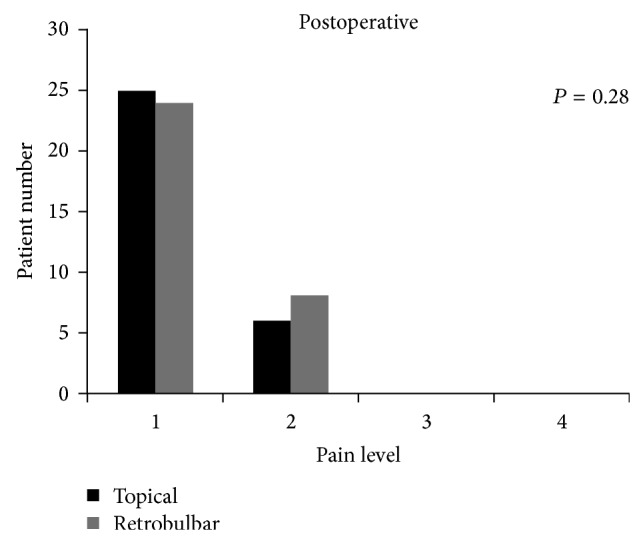
The pain scores recorded for postoperative period.

There was more discomfort in patients in Group 2 while anesthesia was administered (Group 1: 1.0 ± 0, Group 2: 2.3 ± 0.7, *P* = 0.0001, Figure 1). Between the two groups the level of pain during surgery (Group 1: 1.4 ± 0.5, Group 2: 1.5 ± 0.5) was noted. There was no significant difference between two groups peroperatively (*P* = 0.85) and it was shown in Figure 2. There was also no significant difference between two groups postoperatively (Group 1: 1.2 ± 0.4, Group 2: 1.3 ± 0.4; *P* = 0.28, Figure 3).

In Group 2, patients did not encounter any of retrobulbar injection needle-related complications except for one patient who developed conjunctival chemosis minimally. This complication was not observed in Group 1 patients. All patients remained conscious and communicative during the procedure. Additionally, none of the patients required additional retrobulbar, peribulbar, or sub-Tenon anesthesia. For 8 patients (25.8%) in the topical group and 12 (37.5%) in the retrobulbar group, posterior vitrectomy was combined with phacoemulsification and intraocular lens implantation (Table 3). Argon laser photocoagulation was performed in all patients of the topical and retrobulbar groups. After vitrectomy, 1300-centistoke silicone oil was used in 22 (71.0%) patients in the topical group and 20 (62.5%) patients in the retrobulbar group. Sulfur hexafluoride gas was used in 8 (25.8%) and 10 (31.2%) patients in Groups 1 and 2, respectively (Table 3). The distribution of tamponades and use of laser also did not differ significantly between two groups (*P* > 0.05). Minimal subconjunctival escape of gas was noted in one case; and that resolved spontaneously and there was no hypotony. Conjunctival closure was done in 5 and 4 cases in Groups 1 and 2, respectively, in which we observed silicon oil escape. No other significant complications were noted in patients.

In general, all patients communicated well during all procedures. Most of the patients reported Grade 1 pain in the majority of the surgery. In Group 1, worst pain was experienced during initial trocar insertion, endolaser, and cannulas removal. In Group 2, worst pain was experienced during the retrobulbar injection and endolaser.

The surgeon noted squeezing of the eyelids during surgery in 2 patients in the topical group and 1 in the retrobulbar group, but the degree of squeezing did not interfere with the surgery. No patient had inadvertent movement of the eyeball during surgery and there was no complication related to this condition.

Mean surgical time was 47.1 minutes (range 35–73) in Group 1 and 49.2 minutes (range 34–98) in Group 2. The difference was statistically significant (*P* < 0.05).

At the follow-up period, all the surgeries were successful anatomically (e.g., holes were closed, retinas were attached, and vitreous haemorrhages were clear).

## 5. Discussion

Traditionally, vitreoretinal surgeries are performed under general anesthesia. Today, local anesthesia is being used for most of vitreoretinal surgical procedures. As known, many complications have been reported with injection anesthesia, including globe perforation [14–16], injury to optic nerve [17], cranial nerve palsies [18], restrictive strabismus [19], diplopia [20], ptosis [21], retinal vein and artery occlusion [22, 23], and seizures and cardiorespiratory arrest [24, 25]. Mahajan et al. reported that injection delivered in wrong plane can present as significant conjunctival chemosis, which ultimately hinders conjunctival displacement and later conjunctival migration for sutureless port closure [13]. According to our experience when significant conjunctival chemosis occurred during the 23-gauge sutureless surgery, cannulas were raised by edematous conjunctiva from sclera and that can cause choroidal detachment. Lid edema or ecchymosis noted on first postoperative day can be cosmetic concern to the patient [13]. In this study, this needle-related complication was not observed in Group 1 patients.

Previous studies have demonstrated that topical anesthesia can be safe for cataract surgery [5, 7] and some vitreoretinal procedures [5–7, 26]; however, its effectiveness had not been compared directly with retrobulbar injection. The primary outcome of our study was to examine the intraoperative pain control and efficacy of topical anesthesia. This was assessed by interviewing the patients and the attending surgeon. All of the surgeries of the study were made by the same surgeon which eliminated the possible confounding factors which could cause different levels of pain during surgery with different techniques. While designing the study, the distribution of number of cases for indications of posterior vitrectomy was balanced by an experienced surgeon (LK). Additionally the number of cataract surgeries and the surgical time for Group 1 and Group 2 were not significantly different between two groups, which eliminated possible additional confounding factors as discussed in the literature [9–11]. Although the mean surgical time between groups was statistically significant, it was clinically insignificant.

The patients rated the severity of intraoperative pain during administration of anesthetic, peroperatively and postoperatively. As expected, pain sensation during administration of anesthesia was significantly higher in retrobulbar group than in topical group. Of the 32 patients in the retrobulbar group, 46.9% reported the pain during the injection of the anesthetic material as the most painful step of the surgery. Although we might think that less discomfort or pain occurs during surgery with retrobulbar injection, the results of our study showed the fact that both retrobulbar and topical anesthesia provided equivalent pain control during all stages of operation served as an independent verification of the results obtained by interviewing the patients. Bahçecioglu et al. [8] compared the topical and retrobulbar anesthesia in cases undergoing posterior vitrectomy with 20 G technique without scleral buckling procedures. They revealed the highest pain especially during conjunctival opening, the creation of pars plana sclerotomies, external bipolar cautery, and conjunctival closure. In our study, as we used 23 G technique, we did not make any conjunctival opening or external cautery. In our study we found that Group 1 worst pain was experienced during initial trocar insertion, endolaser, and cannulas removal, and Group 2 worst pain was experienced during the retrobulbar injection and endolaser.

None of the patients in both groups received supplemental IV medication which further validates our conclusion that topical anesthesia and retrobulbar anesthesia are both controlling pain during posterior vitrectomy and are equally efficacious.

We excluded patients who had previous posterior segment surgery from the study because conjunctival scarring from those procedures makes manipulations difficult and results in a bias in such patients. Although Yepez et al. [6] demonstrated that topical anesthesia could also be safe for scleral buckling procedures, using intravenous midazolam and fentanyl citrate, we excluded the patients who needed scleral buckling, because these manipulations could cause unbearable pain.

To achieve akinesia, the nerve supply to the extraocular muscles should be inhibited which is generally regarded as desirable for safe intraocular surgery which is not achievable by topical anesthesia. Intraocular manipulation would be expected to be uncomfortable for patients with topical anesthesia. The presence of ocular motility during vitrectomy under topical anesthesia may seem to be a disadvantage because of iatrogenic complications, such as retinal tearing or hemorrhage as intraoperative eye movements may develop. However, intraocular instruments positioned through the pars plana helped the surgeon to steady the eye and to prevent sudden eye movements. The presence of ocular motility during surgery may even be helpful in that the surgeon can ask the patient to look to the intended side which can be helpful especially in cases with retinal detachment while we need excessive vitreous base clearance. We had no case of iatrogenic complications due to sudden movement of the eyeball during the procedure. These findings are compatible with previous studies [8].

Mahajan et al. reported that they specially avoided cases of macular surgeries like macular holes and epimacular membranes but two cases required epiretinal membrane peeling and membranes were peeled successfully. In our study we performed in 2 cases epimacular membrane peeling, in 1 case macular hole surgery, in 4 cases epiretinal membrane peeling in vitreous hemorrhage and proliferative diabetic retinopathy, and in 4 cases internal limiting membrane peeling in Group 1. We did these macular surgery procedures without any complication in topical group. Previously, many authors have reported using topical anesthesia for such cases [6] but in this study to mention the reliability of macular surgery there is a need for a greater number of patients.

Topical anesthesia obviously eliminates the risk of globe perforation, retrobulbar hemorrhage, damage to the optic nerve, and significant conjunctival chemosis. The topical anesthesia technique appears to provide acceptable analgesia during surgery, wears off rapidly after surgery, and does not interfere with the patient's ability to blink, see, or move the eye [6]. In the present study we confirmed these observations. Patients were able to follow commands, and movement of the eyeball was controlled by the surgeon through the surgery with the use of intraocular instruments. Communication with patients was essential for the successfully topical technique.

In conclusion, topical anesthesia is useful for posterior vitrectomy needing no scleral buckling procedure. Topical anesthesia and sutureless surgery are comfortable alternatives, especially in selective cases including outpatient situations and in those patients with high expectations of the surgery.

## Figures and Tables

**Table 1:** Visual analogue pain scale (VAPS).

Pain level	Description
Grade 1	No pain or discomfort
Grade 2	Mild pain and discomfort
Grade 3	Moderate pain and discomfort
Grade 4	Severe pain and discomfort

**Table 2:** Baseline patient characteristics and indications for vitrectomy by treatment assignment.

	Anesthesia	
	Topical	Retrobulbar
*n*	31	32
Sex-number (%)		
Male	19 (54.2)	21 (55.2)
Female	16 (45.8)	17 (44.8)
Age (years)		
Mean	59.3	59.5
Range	47–71	43–76
Indications for PPV		
Vitreous hemorrhage	12	14
Proliferative diabetic retinopathy	8	6
Rhegmatogenous retinal detachment	7	8
Dislocated crystalline or intraocular lens	5	5
Epimacular membrane	2	3
Macular hole	1	2
Duration of surgery (minutes)		
Mean	47.1	49.2
Range	35–73	38–82

**Table 3:** Surgical properties by treatment assignment.

	Anesthesia	
	Topical *n* (%)	Retrobulbar *n* (%)
Cataract surgery	8 (25.8)	12 (37.5)
Tamponade		
Sulfur hexafluoride	8 (25.8)	10 (31.2)
Silicon oil (1300 cs)	22 (71.0)	20 (62.5)
No tamponade	1 (3.2)	2 (6.25)
Conjunctival closure	5 (16.0)	4 (12.5)
